# Do Personal Differences and Organizational Factors Influence Nurses’ Decision Making? A Qualitative Study

**DOI:** 10.3390/nursrep11030067

**Published:** 2021-09-17

**Authors:** Rana Alaseeri, Aziza Rajab, Maram Banakhar

**Affiliations:** 1 Regional Nursing Administration, Hail City 55425, Saudi Arabia; 2 Public Health Nursing Department, King Abdulaziz University, Jeddah 21577, Saudi Arabia; aaarajab@kau.edu.sa (A.R.); ahbbanakher3@kau.edu.sa (M.B.)

**Keywords:** nurses’ decision-making processes, factors influencing nurses’ decisions, naturalistic decision-making approach, managerial decision, clinical decisions

## Abstract

Decision-making processes (DMPs) can be altered by several factors that might impact patient outcomes. However, nurses’ views and experiences regarding the multitude of personal and organizational factors that may facilitate or inhibit their decision-making abilities have rarely been studied. Purpose: To explore the personal and organizational factors that influence nurse DMPs in clinical settings at Ministry of Health hospitals (MOH). Method: A qualitative research design was conducted. A purposive sample of 52 nurses was recruited from general and critical wards in two major Ministry of Health hospitals in Hail, Saudi Arabia. A total of eight focus groups (semi-structured interviews) were conducted to elicit participant responses. Results: In this study, the personal differences covered nurses’ experience, physical and psychological status, autonomy, communication skills, values, and cultural awareness. Organizational factors included the availability of resources, organizational support, workload, the availability of educational programs, the availability of monitoring programs, and the consistency and unity of policies, rules, and regulation applications. Conclusions: The major contribution of this study is the comprehensive illustration of influential factors at both the personal level and the organizational level that impact DMPs to achieve desired outcomes for patients and health organizations. This study utilizes a framework that could explain the nature of nurse DMPs.

## 1. Introduction

Globally, nurses’ decisions have vital implications for the safety and quality of patient care outcomes [1]. Numerous researchers have revealed that a nurse’s decision-making process (DMP) is a complex process surrounded by potentially competing factors embedded in several personal, patient-related, and organizational factors [2,3,4]. The quality of nurses’ decision-making for patient care is varied and unique depending on their knowledge, experiences, and skills [5,6]. For instance, Wu et al. (2016) found a notable relationship between nurses’ prior clinical experience and efficient patient care decisions [7]. According to Salami et al. (2017), experience builds the confidence and decisional awareness of nurses. They found that expert nurses had the power to make non-nursing decisions and possessed the ability to question physicians about their clinical decisions and diagnoses [8]. These traits were absent in less experienced nurses in critical care departments [8]. Nurses’ cognition and attitude, familiarity with patients, and experience with adverse events (e.g., due to the possibility of the accidental extubation of ICU patients, they may be more likely to use physical restraints) vary [9].

Moreover, nurses’ knowledge and education level have a major effect on decision making. Bjørk and Hamilton (2011) indicated that nurses can demonstrate advanced reasoning skills in clinical decisions if they have a bachelor’s degree [10]. These results seem to be consistent with other research that found that nurses with advanced degrees were able to make more effective decisions by building a theory base to guide their DMP [11]. Nurse education was one of the resources that influenced the decision to activate a rapid response team (RRT) [12]. It was also proven that practical training advanced nurses’ knowledge and skills and enabled them to make effective decisions [3].

Nurses use situational awareness to make the right decisions and predict desirable practices of the patient care continuum in the hospital [13]. Cappelletti et al. (2014) found that nurses’ knowledge and situational awareness would support making the right clinical decisions through gathering previous information and bringing it to bear on the current situation [10,14].

Nibbelink and Brewer (2017) revealed that autonomy and self-confidence in nursing interventions contribute positively to clinical decision making [15,16,17]. Dorgham (2013) found a positive relationship between a nurse’s years of experience and decision-making autonomy. A higher decisional autonomy level was found in nurses who had been working for 10–20 years [18]. Indeed, in less complex environments where nurses have control over practice, collaboration, and effective communication about patient care, nurses’ clinical decision-making quality was enhanced [19]. Moreover, Melin-Johansson et al. (2017) found that there was a clear correlation between intuitional decisions in clinical areas and nurses’ knowledge, experience, and working environment [20]. Expert nurses have higher intuitive decision-making abilities and manage situations more confidently than novice nurses, according to the surrounding environment and drawing on experience [21,22].

Researchers have attempted to evaluate the interaction between organizational factors and nurses’ DMPs. These factors were reported as the characteristics of the surroundings and environmental elements such as patient volume, critical care units or patients with complex medical conditions, time aspects, stressors, room features, and the setting’s structure [4,23]. Cappelletti et al. (2014) asserted that the context in which the situation occurs could impact nursing judgment and decision-making [14]. Moreover, Merrick, Fry, and Duffield (2014) found that collegial support and professional relationships have a notable influence on nurses’ decision making, which eventually alters the desired organizational and patient care outcomes [24]. Peer learning, expert consultation, and the leader’s approachability during patient care emergencies were found to be key factors affecting nurses’ decision making [25]. Trusting relationships and collegial support in clinical areas enhanced nurses’ advancement, resulting in more effective decisions, positive patient care outcomes, and nurse retention [3,25,26,27,28].

Nurses often use different decisional tools, such as hospital protocols and guidelines, to manage patient care. Several examples are seen, such as policies for maternal and neonatal care tools in the delivery room [13], and drug administration protocols to prevent errors in the medical and surgical departments [29,30]. Additionally, organizational weaning protocols are one of the critical factors that influence the decision to wean patients off of mechanical ventilation [28]. Patient-centered care focuses on the effects of human and non-human resources on nurse decision-making. For instance, adequate staffing, equipment, beds, supplies, ambulance services, time, and management support can impact a nurse’s ability to make patient-centered decisions [13]. Lane and Harrington (2011) highlighted how nursing shortages lead to increased workloads, which is considered a significant factor in the decision to use physical restraints [9]. Oduro-Mensah et al. (2013) confirmed that nurse decision making could be developed through the Internet, social networking, and the use of phone calls and text messaging with experts during emergencies [13].

Although many studies present findings on the different paradigms used by nurses in decision making, there remains a gap in the international nursing literature as to how nurse managers go about making decisions in clinical settings. Additionally, the number of researchers studying DMP among nurse managers was limited. Moreover, the researchers were unable to locate any previous studies conducted in Saudi Arabia that thoroughly explain the DMPs and factors that influence nurse and nurse manager decision making. Therefore, this research aimed to answer two questions: “what are the personal factors that influence the decision-making process in nursing practice?” and “what are the organizational factors that influence the decision-making process in nursing practice?” The findings of this study revealed a gap in knowledge and evidence about nurse and nurse manager perceptions of the DMP and its essential factors in the Saudi Arabian context. More studies to explore and categorize nurses’ DMP and the various factors influencing it are highly recommended [13]. Therefore, this study emphasizes significant issues confronting the nursing field, at Ministry of Health hospitals in Saudi Arabia in particular, and worldwide. This could lead to some purposeful recommendations for educational and clinical organizations to further prepare, evaluate, and assist in nurse decision making.

## 2. Naturalistic Decision-Making Theoretical Framework

This research paper utilized the NDM framework because of its relevance to nurses’ natural hospital environment practices. The use of NDM in the exploration of decision-making in decision-makers’ real settings was supported by several studies related to the nursing profession [6,15,26]. This study included various factors found to be essential to the NDM framework, including the decision-makers’ factors, factors linked to specific tasks, and environmental factors [6].

The personal factors in the NDM have a combined role and include the decision maker’s experience, knowledge, confidence level, and situational awareness [6,15]. This study reveals that experience, autonomy, nurse values, and the nurse’s physical status and communication skills are key components influencing the nurses’ DMPs. In the absence of these personal characteristics, nurses’ decision making can be ineffective. Based on the NDM framework, environmental factors include organizational goals and norms, time limits, feedback loops, and multiple players [15], which share similarities with this study’s findings. For instance, workload and time pressures were described as the highest organizational factors that influence nurses’ DMPs [16,31,32]. Nurses utilized the NDM by following hospital policies, guidelines, and goals in order to make appropriate decisions [13,28,29].

The need for research that implements this framework is evident due to the number of critical determining factors identified by the participants in this research, which is relative to those indicated in the NDM.

## 3. Materials and Methods

### 3.1. Design

A descriptive qualitative research design was used in this study. The qualitative research method is descriptive in nature and was chosen because it provides a comprehensive understanding of the study questions through a detailed exploration of the particular phenomena involved [33].

### 3.2. Setting

The study was conducted in Hail in two major hospitals: King Khaled Hospital, which has 280 beds and Hail General Hospital, which has 240 beds. The context of these settings is similar to other hospitals directed by the MOH, so the results of this study could be transferred to other hospitals in Saudi Arabia.

### 3.3. Participants

The study participants were chosen from a large nursing population of nurses and nurse managers working full-time in MOH hospitals in Hail, Saudi Arabia. A typical purposive sample was used, where the participants were handpicked from a typical larger population, as mentioned earlier. All employees met the inclusion criteria, including (1) at least two years’ working experience, (2) being from general and critical care units, and (3) willingness to participate in the study and focus group interviews (FGIs). An introductory letter was sent to the two hospitals for participant recruitment; it gave a brief description of the research and researcher contact information. Interested nurses and nurse managers were asked to respond to the FGI in the two weeks after the posting of the letter. Interview participants were limited to the Hail region to provide a degree of homogeneity within the sample, as all participants practiced in the same context and were licensed according to the same professional standards and competencies to support the credibility of the responses. All 73 potential participants were invited to participate in the qualitative interviews. The final number of volunteer participants who agreed to participate from both hospitals was 52 (*n* = 52), and a further explanation of the research was given before each interview session.

### 3.4. Data Collection

A focus group interview (FGI) was considered a proper technique of data collection for this qualitative study. All FGIs were conducted by the first author to support the data confirmability. Staff nurses and nurse managers were interviewed separately in each hospital. In the FGIs, there was a list of questions (“Interview Guide”) related to the influential factors’ impact on nurses’ clinical or managerial decisions to manage the discussion. The interview guide consisted of semi-structured questions, developed by reviewing the literature and approved by four faculty members. The data collection was from April to July 2018. Face-to-face focus group interviews were conducted in series for the optimal detection of patterns and trends across groups to validate the data [34]. A total of eight focus group interviews were conducted at each hospital. Each hospital had two types of focus group participants: four interviews were conducted with nurses and another four with nurse managers to gather data from two different perspectives to represent the 52 participants who volunteered for this study. Each FGI session lasted 60 min. In this study, the final number of FGIs reflected that no new themes and ideas were being generated, signifying data saturation [34]. The meeting time, date, and location of the FGIs were announced to the participants before the meetings.

### 3.5. Data Analysis

The researcher applied a thematic analysis based on a six-phase thematic analysis framework, which is a systematic and objective means of describing nursing decision making and its influential factors [35]. This method of analysis included the following steps: (1) Become familiar with the data. Audio files of eight interviews were played two times before starting the verbatim transcription by the researcher. The data from the focus groups were reviewed by the researcher at least 10 times to allow her to be fully engaged with the data. (2) Generate initial codes. In this step of the thematic analysis, the transcripts were coded and categorized using words and phrases to decontextualize the data and identify units of meaning from each transcript to be arranged systematically in groups according to the specific classification or particular sections using Microsoft Excel. (3) Search for themes. The data were analyzed according to the five main categories to generate themes. (4) Review themes. The data from all focus groups were reviewed and the audit trail was re-examined for patterns or categories. The researcher then re-examined the quotations noted in the audit trail to provide insights into this step in the process. (5) Define themes. Ultimately, in their final refinement, the identified themes were related to the study aim, the nurses’ DMPs, and its influencing factors. (6) Write-up. Writing and reporting the results of the thematic analysis was the last step of the process [35].

### 3.6. Ethical Consideration

This study was approved by the King Abdul-Aziz University Faculty of Nursing, and ethical approval was gained from the human subject protection committee at the Ministry of Health and Institutional Review Board of King Fahad Medical City. Written informed consent forms and a letter of introduction to the study were posted within the nursing office for prospective participants to read. Before signing a consent form, participants were provided with written material about the study and allowed to discuss the content or ask questions about the study, sound recordings, confidentiality, and the accessibility of information to all participants. Participation was voluntary. All participants were aware that withdrawal from the study at any time without consequences was permissible.

## 4. Results

Staff nurses and nurse managers were asked “what are the personal and organizational factors that facilitate or inhibit your decision-making process?” Detailed and various descriptions were provided by both nurses and nurse managers, and a total of 1492 initial codes were extracted. After several reviews, the codes were categorized according to the similarity and proportion. The categories were divided into five main themes, of which this research article will present two: (1) Theme 1: The Influence of Personal Factors on the Nursing Decision-Making Process, and (2) Theme 2: The Influence of Organizational Factors on the Nursing Decision-Making Process.

### 4.1. The Influence of Personal Factors on the Nursing Decision-Making Process

In this section, staff nurses and nurse managers highlighted several personal factors that affect their DMP (Table 1). 

Nurses often work in circumstances that are influenced by their physical or psychological status, which admittedly affects their DMP. Most of the participants emphasized that these statuses could tend to degrade a nurse’s cognitive functioning, which makes it difficult to accurately analyze the information on decision making.

“If we are sick or not feeling well or when we face some personal problem that we encounter everyday it will put us under pressures and stress such as when someone in our family, our husband or our children, is admitted in the hospital; of course, our mind will be with them only. If we cannot focus, we cannot decide; it will reduce our chance of making a good decision and affect our decision-making process.”

S.N.

Furthermore, staff nurses and nurse managers believed that a lack of communication skills, such as a language barrier between nurses, would hinder their DMP. It would lead to misunderstandings and minimize the quality of the nurse’s care and the effectiveness of their decisions:

“Miscommunication will lead to poor decision making. Because I am non-Saudi, I cannot explain many things to the patient; the language barrier is a problem. I try to speak Arabic, but still the patient has many more doubts about the treatment, instructions, tablet, or injection side effect. They cannot understand me, and I can’t decide anything without patient permission.”

S.N.

Another personal factor reported by the study participants was personal values, beliefs, and cultural awareness; this factor plays an integral part in nurses’ choices, actions, and way of making decisions. Nurses have mentioned that their familiarity with different values, religions, and cultures is what enabled them to understand others’ choices and accommodate them in nursing practice.

“Patients’ beliefs also affect the decisions that we are going to make, because this is not our country; this is Saudi Arabia and there are different traditional cultures and religious beliefs that affect our decision making. The patient after surgical operation will put herbal liquid with a strong smell around their nose, thinking that would prevent inflammation at the surgical site. So, I correct this idea with all respect for his decision and continue with the other nursing care decisions.”

S.N.

The participants paid attention to the values of privacy, fairness, and equality as ethical principles involved in nurses’ actions.

“We should give female patients the right to privacy in our decisions. It’s a part of our values, … even during a life-saving situation.”

N.M.

“I have to respect patients’ and staff nurses’ religion. If they are Muslims or Christians, I have to respect them when dealing with them and deciding on their care.”

N.M.

In addition, previous experience is considered a major factor that can impact a nurse’s DMP. Many participants explained how previous clinical experience caused their DMP to be more accurate and faster due to recurrent exposure to a situation.

“Experience will facilitate faster decision making because we would know already what type of decision we need to take for any procedure. For example, the code blue: the first time you attend a code blue, you don’t know anything about it. But if you attend the code several times, then you know what to do.”

S.N.

Staff nurses and nurse managers emphasized that gender differences have an impact on Saudi female nurses’ performance and DMPs due to sociocultural norms and Islamic laws. In this context, female nurses experienced difficulty providing comprehensive patient care to male patients, which could be an obstacle to making good clinical decisions.

“The gender is really affecting Saudi nurses’ decision making during patient care, because they have some limitations coming from their strict culture and beliefs. We have to respect each other’s cultures and religions when deciding about some clinical procedures and assign a non-Saudi nurse to do the male patient care.”

S.N.

Furthermore, the freedom and authority to make clinical or managerial decisions and actions appeared to be connected to their autonomy in nursing practice.

“Here in this hospital, we are allowed to make an independent decision without asking for approval from anybody. We can decide effectively and independently, because we always weigh the results of any situation [and are determined] not to harm the patient or staff nurse or even ourselves and, of course, not to break hospital policies.”

S.N.

### 4.2. The Influence of Organizational Factors on the Nursing Decision-Making Process

The data from the focus group interviews were categorized into six different subthemes presenting the organizational influencing factors that facilitate or inhibit nurses’ DMP (Table 2).

Workload inhibits accurate interventions and patient care choices. In this context, nurses highlighted various issues that increase their workload, which inhibits their ability to make effective decisions, such as a high patient–nurse ratio, doing non-nursing jobs, and interruptions.

(1) High patient ratio:

“Workload resulting from the incorrect nurse–patient ratio … we are sometimes in charge of nine patients each … it is very difficult to think about good decisions or to think about the outcomes of our decisions.”

S.N.

(2) Doing non-nursing jobs:

“Taking over others’ responsibilities affects our nursing decisions, because when some doctors are asking nurses to insert a NGT, it’s taking our time; it’s very difficult to focus on what we need to decide about our [own patients’] care, and we will rush our care to finish on time.”

S.N.

(3) Distractions and interruptions:

“If we are in a place where we were interrupted, and there are many people around us like patients’ relatives, students, and new nurses asking for explanations; or even the alarm system in the hospital is creating noise—all of this affects our decision making and will add to our workload. It’s not easy to decide in an area where we are being distracted by other people.”

N.M.

Moreover, the availability of resources (human and non-human) was considered an important common factor that influences all nurses’ clinical and managerial decisions. The participants highlighted the challenge of nursing shortage that results from nursing absenteeism and nurse performance.

“If we have a high patient population and fewer staff nurses or nurse absenteeism, it means it is difficult to make good care decisions for the patients. It will be very hard to decide how to distribute staff nurses to all patients.”

N.M.

Additionally, the majority of participants emphasized that the shortage of equipment and supplies was an obstacle for making efficient patient care decisions.

“Sometimes we don’t have enough medical supplies, … [so] we cannot give the proper care to the patient. We are deciding to use a heavy pack with cotton on the patient and that cotton maybe will go inside the wound, which makes it very difficult for the wound to heal. For a simple dressing, we are using the big gauze. Insufficient supply will affect our work and decisions and patient care results.”

S.N.

Technology improves the quality of the nursing DMP, as was highlighted by the participants, as they indicated that utilizing technology for patient records and treatment choices and results would facilitate care decisions.

“Before we are receiving the manual report of lab investigation results. Now it will not be missed because we can check them in the computer by the patient’s file number. So, the technology makes our decision faster and it is easy to choose correct actions depending on the results.”

S.N.

Importantly, study participants perceived the need to acquire support from both nursing and hospital administration to increase a nurse’s ability to make the right decisions, as stated by some staff nurses.

“Good support from the organization helps a lot in decision making. Because if we do something wrong but have support and correction from the nursing director or supervisors and hospital administration, we will be confident that we can make good decisions next time.”

S.N.

In addition, nurse managers got support from expert nurses in the unit to help with problems and decision making:

“Even though I am a head nurse, still, in some cases, I will ask some experienced nurses who have previous experience from another unit about their way of dealing with and deciding about the unit’s problems.”

N.M.

Additionally, consistency in the application of policies and procedures and the availability of clinical guidelines enhances patient care decisions:

“We have unity in the policy and we follow the clinical guidelines or protocols to guide us in our decisions, like in the management of hypokalemia … we can decide how much potassium and what IV fluids we should give to the patient.”

S.N.

Moreover, noncompliance with hospital policies and rules by some of the other health team members might cause a dilemma for nurses seeking to make the right decisions and could affect patient care and safety.

“The system is here, but the problem is they are not following the system. Like in changing the central line: it should be every 14 days, but doctors won’t; they will say it is still intact. So, how can you give the medication; it is against the system, but at the same time, we can’t refuse a doctor’s order. The nurses don’t know who is responsible for that kind of decision.”

S.N.

Importantly, a floating system within the organization might inhibit the nurse DMP, which may reduce the quality of nursing care. Floating to another department without receiving proper training causes stress, disorganization, and a feeling of discomfort, even for an expert nurse. This may result from unfamiliarity with a different patient care situation, altering the routinized decision making and various unit protocols.

“When I am pulled out to another department that is very different from my department and unfamiliar to me, the nurses in that department are not always available or helpful. So we are the ones who make all the decisions and take the responsibility; in that situation, we are helpless and stressed and so make hesitant decisions.”

S.N.

Furthermore, continuous monitoring programs have been designed to facilitate nursing professional development and clinical performance. In this context, many nurse managers and staff nurses agreed that activating the nursing competency program had provided them with better learning opportunities and enhanced their ability to make the right decisions.

“Yearly, we must update our unit-specific competencies and, of course, after the competency evaluation, we feel confident that we have the right skills. Our decisions are at excellence level and we get good comments and feedback.”

S.N.

## 5. Discussion

The majority of participants in this study reported that they often work in stressful and challenging working conditions, including sickness and stress, which affects their decision-making ability. Indeed, the participants believed that, in these conditions, it is challenging to understand the environment and the patients’ cues. These results corroborate previous work that examined the effect of these psychological and physical situations on nurses’ decision-making abilities [3,36]. Ham et al. (2017) asserted that fatigue/tiredness is considered a physical condition that can reduce nurses’ decision-making abilities. Due to fatigue, nurses were stressed out about making rapid patient decisions [3]. These findings are consistent with data obtained from the USA by Shirey et al. (2013), who highlighted how frequent exposure to stressful situations might negatively impact nurse managers’ health and DMP, which may threaten other professionals, patients, and organizational outcomes [36]. These findings provide insight into the important influence of these statuses, which could degrade nurses’ cognitive functioning and make it difficult to analyze information for accurate decision making.

In addition, many participants reported that the language barrier reduced their competence in terms of communication skills and decision-making ability. This is consistent with the viewpoint of Irish nurses and midwives, who stated that nurses rely on their professional judgment when managing decisions on new tasks or in new roles [37]. Nibbelink and Brewer (2017) also concluded that nurses’ communication skills in reporting clinical events were associated with their effective decision making and facilitated the determination of needed interventions and the management of a patient’s condition [15]. Further research should be undertaken to investigate the relationship between managerial/clinical skills and nurses’ decision making.

Prior studies have noted the importance of personal values, beliefs, and cultural awareness in nurses’ choices, actions, and ways of making decisions. Many nurses in this study mentioned that their familiarity with different values is what enabled them to understand others’ choices and accommodate them in nursing practice. This finding reflects the study of Lovering (2008), who also found that, in Arab culture, and specifically within the hospital setting in Saudi Arabia, non-Muslim nurses’ and Arab Muslim nurses’ DMPs were affected by values, spiritual beliefs, and sociocultural requirements [38]. On the contrary, inconsistent results were found by Almutairi (2012), who noted that some nurses within the multicultural environment of a Saudi hospital struggled to achieve cultural competence in patient care [39]. He found that nurses had various difficulties in meeting the patients’ cultural and spiritual needs while trying to achieve high-quality care decisions and actions [39]. It seems possible that the findings of this study can guide nurses’ DMPs to achieve comprehensive healthcare for patients, as they should meet the patient’s spiritual, physical, and psychosocial needs. However, this study’s findings present the necessity of further research to provide a detailed understanding of the nature of nursing DMPs within a multicultural environment relating to the educational and organizational role in nursing practice.

In this study, several nurses discussed reliance on previous experience when they were confronted with a challenging patient care decision or unit problems. In addition, nurses in this study linked the higher level of experience to optimal decision-making results. These findings agree with those of another study conducted in Greece, reporting that experienced nurses make more effective clinical decisions than less experienced nurses [11]. Additionally, this finding confirms the results of Nibbelink and Brewer (2017), as they found that nursing experience in clinical areas was the most influential factor in the DMP. They found that experience enhances nurses’ self-confidence and guides nurses to make sound decisions analytically [16]. This suggests an excellent opportunity to put experienced nurses alongside novice nurses in different situations to guide their experience and give them support.

In this study, the results revealed the need for autonomy and safe spaces. The freedom and authority to make clinical or managerial decisions appeared to be connected to autonomy in nursing practice. This finding could explain Dorgham’s (2013) findings, which revealed that Saudi nurses had a higher decision-making autonomy level and greater participation in patient decisions than Egyptian nurses [18]. However, the findings of the current study do not corroborate previous research conducted in China by Wu et al. (2016), who indicated that more flexibility and autonomy and a higher level of empowerment had notable adverse effects, as it might impose psychological stress on nurses and consequently impede their decision-making ability [7]. These results, therefore, testify to the need for further study to illustrate how autonomous nurses differ from non-autonomous nurses in the DMP. Additionally, this brings to the surface the importance of organizational empowerment in nurses’ decision-making autonomy, with a supportive climate that promotes nurses’ decisions in line with patient outcomes.

In this study, participants were highly influenced by various organizational factors that could facilitate or inhibit their DMP. It is interesting to note that the vast majority of responses mentioned the influence of workload on nurse’s DMP. In this context, participants highlighted various issues that increase workload and therefore inhibit their ability to make effective decisions, such as a high patient ratio, doing non-nursing jobs, and interruptions. These findings were also reported by Lane and Harrington (2011), who found that the workload and nursing shortages had a significant effect on nurses’ decisions when caring for elderly patients [31]. This was similar to the findings of Ham et al. (2017), who noted that heavy workloads were identified in the nursing literature as a barrier to sound decision making, resulting in insufficient time to make adequate patient care decisions. Indeed, these important findings were not very encouraging and indicated that the impact of the workload on nurses’ DMP should be taken into consideration [3]. Nurses and nurse managers cannot make effective decisions without adequate resources, a proper nurse–patient ratio, and a supportive structure to assist with their large workload. It is the responsibility of the organizational administration to reduce the workload and facilitate the effectiveness of the nurses. Moreover, measuring the real nursing workload using the activity-based method or the dependency-based method is a prerequisite to an understanding of adequate staffing levels and high-quality nursing care.

This study echoes evidence from previous similar studies examining decision making that concluded that a positive work environment with adequate human and non-human resources enables nurses to be effective decision makers and to achieve high levels of care [2,3,16,21,40]. Additionally, in this study, all the participants mentioned the challenge of nursing shortages. Participants believed that the nursing shortage caused by absenteeism, increased patient numbers, and an insufficient number of nurses in the unit inhibited the DMP. Furthermore, nurses and nurse managers indicated that the nursing shortage leads to tasks being left undone, an insufficient quality of care, and affects patient safety. This finding was similar to Lane and Harrington’s (2011) results, as they concluded that a nursing shortage had a significantly negative impact on nurses’ decisions in elderly patient care [31]. The findings of Cho et al. (2016) in South Korea showed that nursing shortages and worse nurse–patient ratios were significantly associated with poor patient safety, poor quality of care, overtime being worked, and care tasks being left undone [41]. The findings of this study can support policymakers’ decisions on human resources management by prompting them to secure and maintain adequate numbers of nurses and improve retention measures [41]. Stakeholders should pay attention to nurse staffing levels, the nurse–patient ratio, and practical things that may decrease the workload to reach optimal levels of care and patient safety.

Additionally, the majority of participants emphasized that a shortage of equipment and supplies was an obstacle to making efficient patient care decisions. In fact, nurses would accommodate this issue by deciding to use lower-quality materials to deliver the required patient care. This finding corroborates Oduro-Mensah et al.’s (2013) results from the Ghanaian context, which revealed that, as nurses interact with patients, they constantly make decisions focused on patients’ needs, which require adequate staffing, beds, equipment tools, supplies (e.g., oxygen cylinders), and referral vehicles and ambulance services [13]. Thus, clearly, these findings support the idea that the availability of human and non-human resources would improve compliance with standards and assist nurses in making accurate decisions.

The current study confirms that organizational support is a factor facilitating the DMP in many aspects of nursing. The participants perceived that they need to acquire support for their decisions from different levels, including the nursing and hospital administration, nursing colleagues, and other health team members. This finding seems to be similar to research conducted by Merrick et al. (2012), who found that collegial and supervisory support enhanced organizational possibilities among Australian nurses, allowing them to practice effective decision making and, therefore, ensuring positive patient care outcomes and increased nurse retention [25]. Additionally, another study by Chisengantambu et al. (2018) revealed that effective decision making requires organizational support, which impacts a nurse manager’s performance, their perception of being appreciated and valued, and the creation of a positive environment [42]. It can thus be suggested that nurses’ and nurse managers’ performance is improved by the continuous support offered from all organizational levels. Additionally, it is important to note that the influence of organizational support in DMPs has great implications for building trust, enhancing nurses’ commitment, and creating a positive work environment to achieve the organizational goals.

The importance of consistency in the application of policies and procedures was mentioned in 19 different statements. The participants believed that hospital policies, roles, and guidelines enhanced patient care and facilitated healthcare members’ decision making. This is consistent with Oduro-Mensah et al.’s (2013) results, which indicated that hospital protocols and guidelines were used as decision-making aids to manage some situations, such as when nurses were not sure about accurate steps to take in maternal and neonatal care [13]. In accordance with these findings, a previous study conducted in the United Kingdom by Dougherty et al. (2012) found that nurses base their decisions on drug administration on hospital policies and guidelines, which should prevent drug errors [29]. There are several possible explanations for these findings. The importance of policies, guidelines, and rules is already recognized in MOH hospitals in Saudi Arabia, where this study was conducted. However, the consistency of these protocols and their application by all health team members is still inadequate. The findings of our study have shown that certain policies and guidelines are not adhered to, which is linked to potential risks and errors. The noncompliance with hospital policies by some health team members is an important issue. Making employees change their behavior is not just about providing them with training or making more policies and procedures, but also about changing their attitudes towards compliance. Increasing knowledge of the policies is not enough to confirm compliance. Therefore, training that gives proper justifications for the policies, investigating noncompliance, and providing adequate resources and a positive working environment might decrease the bias towards compliance with policies among all health team members. In addition, this finding suggests that up-to-date policies, guidelines, and protocols that support effective nursing DMPs are crucial.

Another important finding of this study is that the availability of continuous monitoring programs designed to facilitate nurse performance had a positive influence on decision making. In this context, many nurse managers and staff nurses agreed that the nursing competency program had provided them with better learning opportunities and enhanced their ability to make the right decisions. This finding corroborates the ideas of Thompson and Stapley (2011), who suggested that interventions should consider individual nurses’ differences to improve the nurses’ professional performance and educational level, which has a major effect on patient outcomes [1]. These results seem to be consistent with other research conducted in the USA that found that nurse managers play a crucial role in terms of strengthening staff nurses’ decision making on patient safety and care quality. That role was associated with continuous monitoring of staff nurses’ performance. Nurse managers conducted frequent formal audits to evaluate staff nurses’ skills and patient outcomes [43]. Our findings revealed a positive association between nurses’ decision-making ability and performance monitoring. These relationships may be explained by the fact that, when there is a system evaluating the outcome of nurses’ chosen actions and providing continuous useful feedback, this guides nurses’ future decisions. Therefore, it is suggested that, in the process of evaluation, more attention should be paid to effective feedback methods to ensure that nurses achieve the organizational goals. Considering the perceptions of evaluators, nurse evaluation will increase the efficiency of these programs.

This research was limited to the northern region of Saudi Arabia, which may not reflect other regions in the country. This places a minor limitation on the transferability of the findings. Even though the information obtained was comprehensive, more studies among different hospitals across Saudi Arabia are needed to increase the possibility of generalizing the findings.

## 6. Conclusions

This research aimed to explore nurses’ perceptions about the key factors that influence the DMP in nursing practice. The research findings indicated numerous factors that may facilitate or inhibit care and decision making, which can be a steppingstone for future worldwide research in this field. The important contribution of this study is its comprehensive representation of decision-making factors at a personal, environmental, and organizational level. It has highlighted the need to strengthen nurses’ positive environment and has utilized a framework that could explain nurses’ DMP, including various factors affecting their decisions. Further educational initiatives and future research to enhance nurses’ ability to integrate effective clinical and managerial decisions into their practice are needed, which would lead to improved patient and organizational outcomes. Additionally, in order to expand the understanding of the DMP, it will be necessary to conduct a further study utilizing nursing decision-making models in order to note similarities and differences between administrative, clinical decisions and other types of decisions, such as ethical decision making. Furthermore, to standardize DMP models in MOH hospitals, it would be useful to construct a unified, standardized decision-making model that considers the influential factors that impact the process. To overcome major challenges, the nursing workforce should be well maintained, educated, and trained to be able to apply essential competencies in patient care.

## Figures and Tables

**Table 1:** Comparison of personal factors’ effect on decision-making process between nurse managers and staff nurses.

Theme	Sub-Theme	Nurse Managers	Staff Nurses	Total
The Influence of Personal Factors on Nursing Decision Making Process	Nurse’s Physical and Psychological Status	9	9	18
	Nurse’s Communication Skills	9	6	15
	Nurse’s Values, Beliefs and Cultural Awareness	3	9	12
	Nurse’s Experience	5	6	11
	Gender Differences	2	4	6
	Autonomy	1	3	4
	Total	29	37	66

Note. Number of statements per sub-theme from staff nurses and nurse managers.

**Table 2:** Comparison of organizational factors’ effect on decision-making process between nurse managers and staff nurses.

Theme	Sub-Theme	Nurse Managers	Staff Nurses	Total
The Influence of Organizational Factors Effect on Decision-Making Process	The Workload	12	13	25
	Availability of the Resources	8	14	22
	Organizational Support	9	11	20
	Consistency and Unity of Policies, Rules and Regulation Application	7	12	19
	Availability of Continuous Monitoring and Evaluation Program	1	6	7
	Total	37	56	93

Note. Number of statements per sub-theme from staff nurses and nurse managers.

## Data Availability

Not applicable.
